# Spectral Asymmetry Induces a Re‐Entrant Quantum Hall Effect in a Topological Insulator

**DOI:** 10.1002/advs.202307447

**Published:** 2024-03-13

**Authors:** Li‐Xian Wang, Wouter Beugeling, Fabian Schmitt, Lukas Lunczer, Julian‐Benedikt Mayer, Hartmut Buhmann, Ewelina M. Hankiewicz, Laurens W. Molenkamp

**Affiliations:** ^1^ Institute for Topological Insulators Am Hubland 97074 Würzburg Germany; ^2^ Physikalisches Institut, Experimentelle Physik III Universität Würzburg Am Hubland 97074 Würzburg Germany; ^3^ Institute for Theoretical Physics and Astrophysics (TP IV) Universität Würzburg Am Hubland 97074 Würzburg Germany

**Keywords:** magnetotransport, narrow‐gap semiconductors, quantum anomalies, quantum Hall effect, topological insulators

## Abstract

The band inversion of topological materials in three spatial dimensions is intimately connected to the parity anomaly of 2D massless Dirac fermions, known from quantum field theory. At finite magnetic fields, the parity anomaly reveals itself as a non‐zero spectral asymmetry, i.e., an imbalance between the number of conduction and valence band Landau levels, due to the unpaired zero Landau level. This work reports the realization of this 2D Dirac physics at a single surface of the 3D topological insulator (Hg,Mn)Te. An unconventional re‐entrant sequence of quantized Hall plateaus in the measured Hall resistance can be directly related to the occurrence of spectral asymmetry in a single topological surface state. The effect should be observable in any topological insulator where the transport is dominated by a single Dirac surface state.

## Graphical abstract

The band inversion of 3D topological materials is intimately connected to the parity anomaly of 2D massless Dirac fermions. The parity anomaly manifests itself as spectral asymmetry, an imbalance between conduction‐ and valence‐band Landau levels at a single surface of the device. This leads to an unconventional re‐entrant sequence in the quantum Hall effect, revealed by magnetotransport experiments in (Hg,Mn)Te.

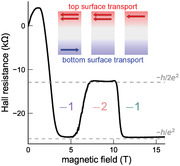

## 1 Introduction

**Figure 1:**
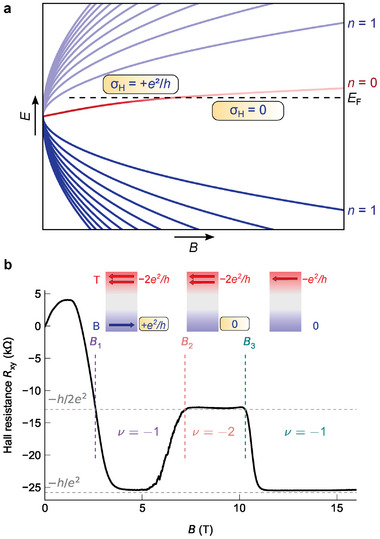
Re‐entrant quantum Hall effect in a 3D topological insulator device due to spectral asymmetry. a) Mechanism of spectral asymmetry. Upon increasing the magnetic field *B*, the band inversion is lost, and the zero Landau level moves from the valence band to the conduction band. The zero Landau level becomes unoccupied and its contribution of *e* ^2^/*h* to the Hall conductance vanishes. b) Observation of the re‐entrant quantum Hall effect in the *p*‐type regime. Upon increasing the magnetic field *B*, the Hall resistance *R*_*xy*_ settles at a plateau at the quantized value of −*h*/*e* ^2^ (ν = −1), followed by a transition to −*h*/2*e* ^2^ (ν = −2) and a re‐entrance to −*h*/*e* ^2^. The schematics (insets) indicate the contributions of top and bottom surface to the Hall conductance. We identify the change in Hall conductance by −*e* ^2^/*h* at the transition from ν = −1 to −2 (at *B*_2_) as the vanishing of the zero Landau level of the bottom surface state, due to spectral asymmetry. The transition from ν = −2 to −1 (at *B*_3_) is an ordinary *p*‐type quantum Hall transition of the massive surface states at the top surface. This data has been taken for $V_{\mathrm{TG}}^{*}=-0.17$ V with bottom gate grounded. We mark the characteristic fields *B*_1_, *B*_2_, and *B*_3_.

Quantum field theory predicts that a single massless 2D Dirac fermion exhibits the parity anomaly.^[^ ^1^, ^2^ ^]^ In a metal, the anomaly induces an ambiguity in the sign of the transverse conductivity in the limit of vanishing magnetic field.^[^ ^2^, ^3^ ^]^ While the connection between the parity anomaly and topological surface states^[^ ^4^, ^5^, ^6^, ^7^ ^]^ has been hinted at extensively,^[^ ^3^, ^8^ ^]^ one can argue that a true observation of the parity anomaly in a condensed matter system, i.e., in the limit of zero magnetic field, is still outstanding. Such an observation is challenging because topological surface states come in pairs and the anomaly can occur only within a single Dirac state.^[^ ^1^, ^2^, ^8^ ^]^ While in the quantum anomalous Hall effect one of the entangled surface states is removed, the effect occurs^[^ ^9^, ^10^ ^]^ only in ferromagnets. Thus, the hysteresis connected with ferromagnetism makes an observation of the anomaly at zero field very difficult. In this work, we report on the experimental observation of a spectral asymmetry in the transport characteristics of the 3D paramagnetic topological insulator (Hg,Mn)Te. The spectral asymmetry,^[^ ^11^ ^]^ a consequence of the parity anomaly,^[^ ^1^, ^2^, ^3^, ^12^ ^]^ induces an anomalous Hall response, i.e., an extra contribution of *e* ^2^/*h* to the Hall conductance σ_H_ from the unpaired zero Landau level (see **Figure** **1**a), when the bulk bands are in inverted order.^[^ ^13^, ^14^ ^]^ In our magneto‐transport experiments it manifests itself as a remarkable sequence of ν = −1, −2, −1 quantum Hall plateaus for increasing magnetic fields. This observation requires the coexistence of topological Dirac surface states and massive (non‐topological) surface states, where the ν = −1 to ν = −2 transition marks the crossing of an unpaired topological zero Landau level with the Fermi level, changing the Hall resistance *R*_*xy*_ from −*h*/*e* ^2^ to −*h*/2*e* ^2^ (see Figure 1b). The parity anomaly should be accessible in any topological insulator device, provided that a single surface can be accessed in magneto‐transport and that the material is of sufficiently high quality.

## 2 Results and Discussion

**Figure 2:**
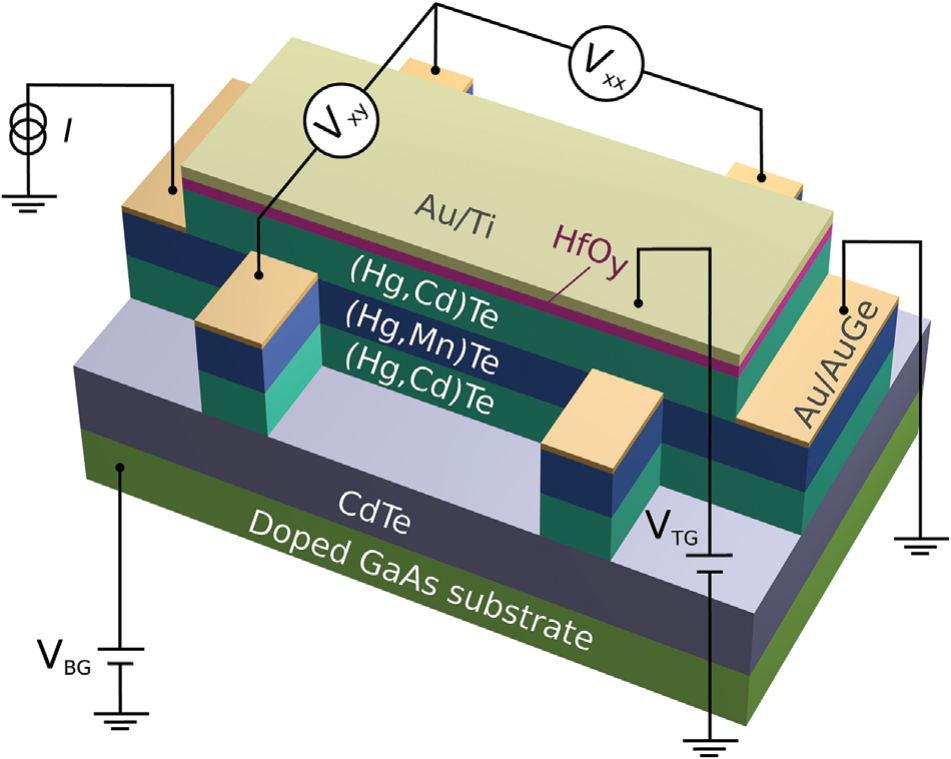
Structure of the Hall bar device. Illustration of the layer structure (thickness not to scale) and the Hall measurement configuration. We indicate the (Hg,Mn)Te topological insulator layer and the (Hg,Cd)Te barriers. The doped GaAs substrate acts as bottom gate and is separated from the bottom barrier by a CdTe buffer. The Au/Ti top gate is separated by a thin insulating HfO_*y*_ layer. The transport measurements are performed through the Au/AuGe Ohmic contacts.

The transport data are obtained for a Mn‐doped 3D topological insulator device of HgTe. A 73 nm thick Hg_1 − *x*_Mn_*x*_Te layer (*x* = 0.017) is grown on a 4 µm thick CdTe buffer layer, sandwiched between thin (15 nm) barriers of non‐inverted Hg_0.3_Cd_0.7_Te (see **Figure** **2**). The lattice mismatch between (Hg,Mn)Te and CdTe provides the required tensile strain to turn the semimetallic (Hg,Mn)Te into a topological insulator with a bulk bandgap of approximately 20 meV.^[^ ^15^, ^16^ ^]^ The Mn‐doping of HgTe is isoelectrical and introduces paramagnetism which enhances the *g*‐factor significantly,^[^ ^17^, ^18^, ^19^ ^]^ and predominantly increases the Landau level splitting already at low magnetic fields of up to 2 T, where the paramagnetic exchange is similar in size as the cyclotron energy. The transport results were obtained from a 600 × 200 µm^2^ sized Hall bar equipped with top and bottom gates (see Figure 2 and Experimental Section). Four additional samples have been investigated with Mn concentrations ranging between 1.1% and 4.4% and layer thicknesses between 64 and 92 nm (see Table S1, Supporting Information). All samples exhibit the same magnetotransport feature discussed below (see Figure S1, Supporting Information).

**Figure 3:**
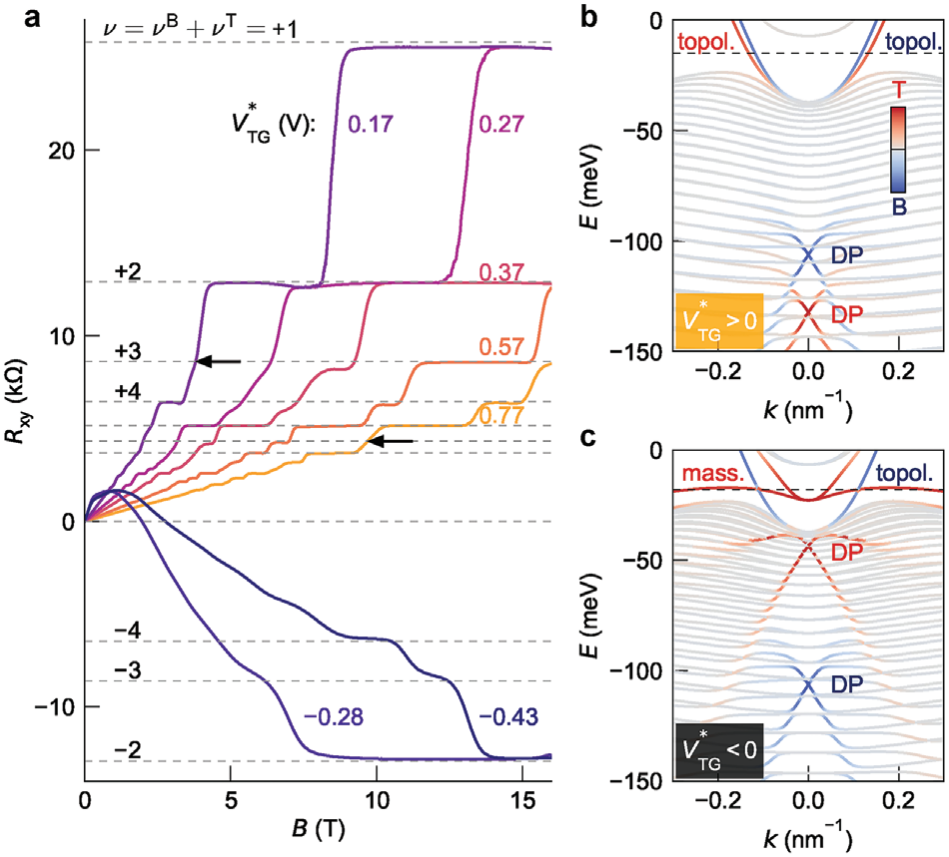
Ambipolar transport and quantum Hall effect in a 73 nm thick (Hg,Mn)Te layer. a) Quantum Hall effect at various $V_{\mathrm{TG}}^{*}$, with bottom gate grounded. Two missing plateaus are indicated by arrows. The quantized values *R*_*xy*_ = *h*/*e* ^2^ν for integer ν are marked by dashed lines. The panels on the right show the band structure from an eight‐orbital *k* · *p* calculation for two distinct scenarios: b) The *n*‐type conducting regime $V_{\mathrm{TG}}^{*}>0$ and c) The *p*‐type conducting regime $V_{\mathrm{TG}}^{*}<0$. We denote the Dirac point (DP), the topological surface states (“topol.”), and the massive surface states (“mass.”) in (b) and (c). The dashed line indicates the Fermi energy. The color code indicates the wave function location: top surface (red), bottom surface (blue), or elsewhere (grey). The *R*_*xx*_ measurements corresponding to (a) are provided in Figure S2 (Supporting Information).

In **Figure** **3**a, we show the low‐temperature (*T* = 120 mK) measurement of the Hall resistance *R*_*xy*_, for various top gate voltages, ranging from −0.43  to +0.77 V and grounded bottom gate (*V*_BG_ = 0). For positive top gate voltages the conductance is purely n‐type, while for negative gate voltages, two carrier types coexist: n‐type carriers dominate at low and p‐type carriers at high magnetic fields. The n‐type carriers are characterized by low density and high mobility, while the p‐type carriers come with a lower mobility but a large density.^[^ ^20^ ^]^ Owing to the high quality of the samples a pronounced quantum Hall effect is observed for positive as well as for negative gate voltages. In order to compare measurements of different samples, the gate voltage is referenced to zero (i.e., we set the effective applied voltage $V_{\mathrm{TG}}^{*}=0$ V) where the appearance of a two carrier type Hall effect becomes observable. Missing steps in the Hall plateau sequence for positive gate voltages indicate the existence of two independent n‐type carrier systems (see for example Figure 3a, ν = 6 for $V_{\mathrm{TG}}^{*}=0.77$ V, arrow). Referring to ref. [15], we identify them as topological surface states related to the top and bottom surfaces.

The appearance of p‐type carriers for negative gate voltages is related to gate voltage induced massive surface states, as is typical for any topological insulator. The gate voltage pulls the non‐topological massive surface states (also known as massive Volkov‐Pankratov states^[^ ^4^ ^]^) out of the bulk valence band.^[^ ^20^ ^]^ The corresponding band structure calculation is given in Figure 3b,c for positive and negative top gate voltages, respectively. This result confirms the observation that for positive gate voltages two massless topological surface states dominate the transport properties (Figure 3b) while for negative gate voltages an additional p‐type massive surface state, located at the top surface contributes to transport (Figure 3c). Note that the high density of states of the valence band (van Hove singularity) pins the Fermi level. For large negative gate voltages, the massive surface state is pulled out of the bulk (cf. Figure 3c), which effectively empties the topological surface state at the top surface, while negative charge still exists in the bottom topological surface state.

The interplay between massive and topological surface states becomes most strikingly visible in the Hall resistance *R*_*xy*_ for top gate voltages between $V_{\mathrm{TG}}^{*}=-0.13$ and −0.25 V. Figure 1b shows the situation for $V_{\mathrm{TG}}^{*}=-0.17$ V. Slightly above *B* = 1 T, the initial positive slope in *R*_*xy*_ becomes negative and a clear ν = −1 quantum Hall plateau develops for *B* > 4 T. Strikingly, for *B* > 5 T, *R*_*xy*_ decreases again and a ν = −2 quantum Hall plateau develops which lasts up to 10 T before it reenters the ν = −1 state. For the following discussion, we distinguish the two ν = −1 states as low‐field and high‐field and define three magnetic fields, *B*_1_, *B*_2_, and *B*_3_, which mark the crossings of *R*_*xy*_ with the ν = −2 line (*R*_*xy*_(*B*_*i*_) = −*h*/2*e* ^2^; cf. Figure 1b).

**Figure 4:**
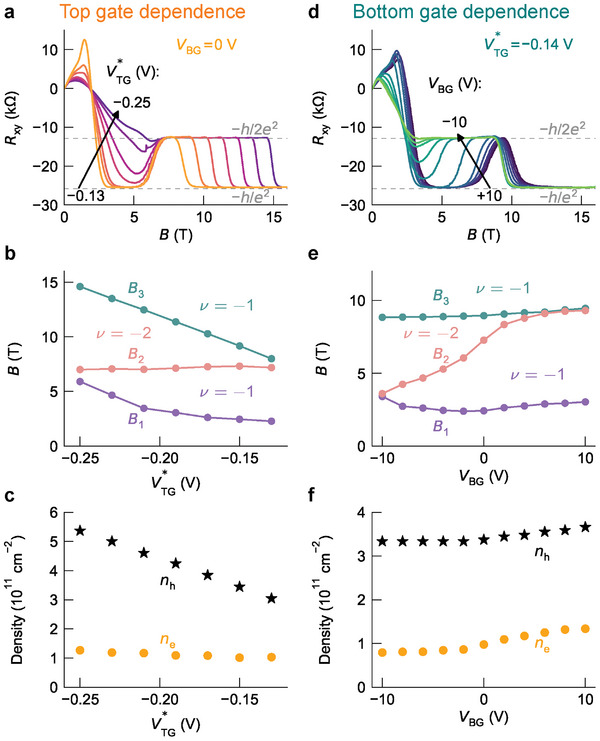
Re‐entrance of the ν = −1 quantum Hall plateau and its dependence on top and bottom gate voltages and carrier densities. Top gate dependence a–c) (the bottom gate is grounded): a) *R*_*xy*_ as a function of magnetic field *B* at various top gate voltages from $V_{\mathrm{TG}}^{*}=-0.13$ to −0.25 V. b) Characteristic fields extracted from (a). We have indicated the quantum Hall filling factors ν between the characteristic fields. c) Electron *n*_e_ and hole density *n*_h_. d–f), Like (a–c), but for bottom gate dependence, at constant top gate voltage $V_{\mathrm{TG}}^{*}=-0.14$ V. The *R*_*xx*_ measurements corresponding to (a) and (d) are provided in Figure S3 (Supporting Information).

To investigate the origin of the re‐entrant behavior, we make use of the fact that our device is equipped with separate top and bottom gates. Importantly, due to screening, the electrostatic gates influence mainly those surface states, which are in closest proximity. **Figure** **4** shows the variation of *R*_*xy*_ as a function of either top or bottom gate voltage (left and right column, respectively), while the other gate is kept at constant voltage. For a variation of the top gate voltage from −0.13 to −0.25 V (Figure 4a), we observe a gradual vanishing of the low‐field ν = −1 plateau, while *B*_2_ remains almost unchanged. At the same time, the onset of the high‐field −1 plateau (*B*_3_) shifts to higher magnetic fields. The deduced characteristic magnetic fields, *B*_1_, *B*_2_, and *B*_3_, are shown in Figure 4b. Analyzing the corresponding carrier densities, we find that the n‐type carrier density (initial Hall slope) remains effectively constant while the p‐type density increases monotonically with increasingly negative top gate voltage (Figure 4c). The behavior is consistent with the idea that the top gate voltage mainly affects the top surface states, increasing the p‐type carrier density *n*_h_ of the massive surface state while the carrier density *n*_e_ of the n‐type top topological surface state is pinned by the van Hove singularity. The situation is opposite for a variation of the bottom gate voltage (Figure 4d–f). Due to the different dielectric constants and the thickness of the insulating layer, the efficiency of the bottom gate is approximately two orders of magnitude less than for the top gate. We find that the low‐field ν = −1 plateau is largest for *V*_BG_ = +10 V and gradually vanishes in the range down to *V*_BG_ = −10 V. In this case, *B*_1_ and *B*_3_ remain constant while *B*_2_ changes strongly, and correspondingly, the n‐type density *n*_e_ exhibits a stronger decrease than the p‐type density *n*_h_.

From the above observations, we conclude that the low‐field ν = −1 plateau is the result of the interplay of quantized Hall conductances for top and bottom surface states. The combined total quantum Hall filling factor, which is observed in the measurement, can be divided into independent contributions from both surfaces: ν = ν^B^ + ν^T^. The component ν^B^ is entirely related to the n‐type carrier density of the topological bottom surface states. The component ν^T^ is controlled by the carrier density of the top surface, which is dominated by the massive p‐type surface state. Its density is correlated with the magnetic field values for *B*_1_ and *B*_3_. At *V*_BG_ = −10 V, the bottom surface state is partially depleted, thus the low‐field ν = −1 is absent in the transport experiment (see also Figure S5, Supporting Information). However, the bottom surface state is most populated at *V*_BG_ = +10 V and the low‐field ν = −1 state has the widest range in magnetic field. We may relate the transition of ν = −1 to −2 and back to −1 to a sequential change of the filling factor for top and bottom surface state as indicated in Figure 1b: For the low‐field ν = −1 plateau, ν^B^ + ν^T^ = 1 + (− 2), the intermediate‐field plateau has filling factors ν^B^ + ν^T^ = 0 + (− 2), and the high‐field plateau has ν^B^ + ν^T^ = 0 + (− 1). Thus, the open question is to identify the transition of the topological surface state from filling factor ν^B^ = 1 to ν^B^ = 0, characterized by *B*_2_ and controlled by the carrier density of the bottom surface state, as coming from spectral asymmetry.

**Figure 5:**
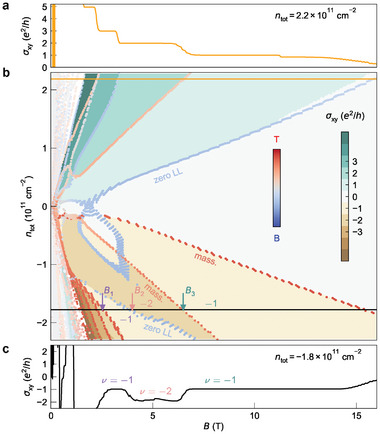
Landau level fan chart reproduced by an eight‐orbital *k* · *p* calculation. b) The color code in the background is associated with the calculated Hall conductivity σ_*xy*_ in unit of *e* ^2^/*h*. The Landau levels are indicated by the dotted dispersions and their color indicates the wave function location: top surface (red), bottom surface (blue), or elsewhere (grey). The vertical axis represents the total density *n*_tot_ = *n*_e_ − *n*_h_, calculated from an assumed Hartree potential *U*_H_ (see Note S4 and Figure S6, Supporting Information for a detailed explanation). The re‐entrant ν = −1 quantum Hall plateau emerges for *n*_tot_ < −1.3 × 10^11^ cm^−2^. The most relevant Landau levels in this re‐entrance are highlighted: “zero LL” for the zero Landau level from the topological surface state and “mass.” for the Landau levels from the massive surface state (at the bottom and top surface, respectively). The “zero LL” appears at multiple densities because the Fermi level depends non‐monotonically on *n*_tot_ due to pinning to the massive surface state (see Note S4, Supporting Information). a) Calculated Hall conductivity σ_*xy*_ at total density *n*_tot_ = 2.2 × 10^11^ cm^−2^ [yellow line in (b)] and c) Calculated Hall conductivity σ_*xy*_ at total density *n*_tot_ = −1.8 × 10^11^ cm^−2^ [black line in (b)]. For Landau fans at these densities, refer to Figure S6 (Supporting Information).

In order to substantiate our assignment of the origin of the re‐entrant quantum Hall effect, we perform Landau level calculations using an eight‐orbital *k* · *p* method. We present Hall conductivity σ_*xy*_ as a function of magnetic field in the range *B* = 0 to 16 T and of the total density *n*_tot_ inferred from the measurement, by varying the Hartree potential *U*_H_ (see **Figure** **5**b and more details in Note S4, Supporting Information). This figure additionally has the color coded information on the location of the carrier system responsible for the indicated Landau level transition (red, top (T) and blue, bottom (B) surfaces, respectively). This method allows us to examine the occupation of each Landau level at a constant total density (gate voltage or Hartree potential) and thus to extract the corresponding filling factors ν = ν^B^ + ν^T^. Two exemplary Hall traces are given in Figure 5a,c for total carrier densities of 2.2 × 10^11^ and −1.8 × 10^11^ cm^−2^, corresponding to $V_{\mathrm{TG}}^{*}=0.1$ V and $V_{\mathrm{TG}}^{*}=-0.2$ V, respectively and indicated by horizontal solid lines in Figure 5b. For positive gate voltages (i.e., *n*_tot_ = 2.2 × 10^11^ cm^−2^, yellow line), a monotonic integer sequence of quantum Hall plateaus appears (Figure 5a; Figure S6b,c, Supporting Information), as Landau levels from the top and bottom surface states are depopulated. Note that the ν = 4 plateau is missing due to the crossing of Landau levels from top and bottom surface states, similar to the phenomenology of the experiments. For *n*_tot_ < −1.3 × 10^11^ cm^−2^ (Figure 5c; Figure S6d,e, Supporting Information) we clearly observe the re‐entrant sequence, ν = −1, −2, −1, comparable with the presented experiment. By examining the wave function profile (|ψ(*z*)|^2^, where *z* is the growth direction) and the orbital character of the Landau levels (see Figure S7, Supporting Information), we establish their origin: the low‐field ν = −1 state is a combination of ν^B^ = 1 from the topological (bottom) surface state and ν^T^ = −2 from the massive surface state at the top surface (see Figure 1b). At *B*_2_, the bottom surface state transitions to ν^B^ = 0 while ν^T^ = −2 remains unchanged.

The transition of ν^B^ = 1 to ν^B^ = 0 represents a single Dirac fermion state in two spatial dimensions and is a signature of the spectral asymmetry related to the parity anomaly known from quantum electrodynamics. This transition occurs when the zero Landau level of the topological surface state crosses the Fermi level in the vicinity of *B*_2_, reducing the Hall conductance by *e* ^2^/*h*, i.e., the total filling factor, ν = ν^B^ + ν^T^, changes from ν = 1 + (− 2) to ν = 0 + (− 2). At this position the zero Landau level changes from the valence band to the conduction band regime, which causes a change of the spectral asymmetry, i.e., the scenario illustrated by Figure 1.

To make this statement more precise, we describe the bottom surface state by the effective Dirac‐like Hamiltonian in the basis {|B; ↑〉, |B; ↓〉}, given by

(1) $$\begin{array}{cc} H_{B} & =C\sigma_{0}+m_{k}\sigma_{z}-A\left(k_{y}\sigma_{x}-k_{x}\sigma_{y}\right)\\ & =\left(\begin{array}{cc} C+m_{k} & -iAk_{-}\\ iAk_{+} & C-m_{k} \end{array}\right) \end{array}$$

where $m_{k}=M-B{\left|k\right|}^{2}$ is an effective mass term which is only present in finite magnetic fields, σ_0_ is the 2 × 2 identity matrix, σ_*x*, *y*, *z*_ are the Pauli matrices in the spin basis, **k** = (*k*_*x*_, *k*_*y*_) is the momentum, and *k*_±_ = *k*_*x*_ ± i*k*_*y*_. The contribution of the spectral asymmetry to the Hall conductance is given by (see Note S5, Supporting Information and ref. [13])

(2) $$\begin{array}{c} \sigma_{xy}^{B}=-\frac{e^{2}}{2h}\left[\mathrm{sgn}\left(B\right)+\mathrm{sgn}\left(M-\frac{B}{l_{B}^{2}}\right)\right] \end{array}$$

where $l_{B}=\sqrt{\hbar /eB}$ is the magnetic length corresponding to the magnetic field *B*. We emphasize that the coefficients M and B also depend on the magnetic field. We assume B<0. The spectral asymmetry manifests itself as a jump of the conductance $\sigma_{xy}^{B}$ from *e* ^2^/*h* (ν^B^ = 1) to zero (ν^B^ = 0), where the effective mass term, the second term in Equation (2), changes from negative to positive. This jump occurs exactly where the zero Landau level crosses from the valence to the conduction band (see Figure 1a). The sign change of the mass gap indicates the transition from inverted to normal band ordering, a fundamental feature of topological materials.

## 3 Conclusion

In conclusion, we demonstrate the observation of a single 2D Dirac system, realized by separate control of the carrier densities on both surfaces of a 3D topological insulator. The re‐entrant sequence of quantum Hall plateaus allows us to unambiguously identify a single 2D Dirac fermion. At this transition from ν = −1 (low‐field) to ν = −2 the contribution of *e* ^2^/*h* from the bottom surface state vanishes, which is direct evidence for the presence of a spectral asymmetry. Importantly, the contribution from spectral asymmetry persists in the limit of zero magnetic field, *B* → 0. This observation is thus a robust signature for a solid‐state analogue to the parity anomaly.

## 4 Experimental Section

### Sample Description

The sample for which the results are shown in the main text is labelled Sample S1. Information on four further (Hg,Mn)Te samples (labelled Samples S2–S5) is provided in Table S1 (Supporting Information). All the devices exhibit a plateau transition from the low‐field ν = −1 to ν = −2 (Figure S1, Supporting Information), similar to the phenomenology discussed in the main text.

The Samples S1 and S2 have been grown on a commercial Si‐doped GaAs substrate that serves as a bottom gate. First, a CdTe buffer layer has been grown on the substrate; see the previous work ref. [16] for more details. On top of the buffer, a “sandwich structure” consisting of a (Hg,Cd)Te barrier has been grown, the (Hg,Mn)Te layer, and another (Hg,Cd)Te barrier. Samples S3 and S4 are similar, but use a CdTe substrate, i.e., without a bottom gate. In Sample S5, the (Hg,Mn)Te layer has been grown directly on the CdTe substrate. The relevant sample parameters are provided in Table S1 (Supporting Information).

The transport results have been obtained from 600 µm × 200 µm sized Hall bars. The samples are equipped with Au top gates (100 nm thick) separated from the rest of the structure by a 15 nm thick hafnium oxide (HfO_*y*_) insulating layer and a 5 nm Ti layer. The ohmic contacts are made of 50 nm AuGe and 50 nm Au.

### Transport Measurement

The magneto‐transport measurements have been performed with low‐frequency lock‐in technique in ^3^He‐^4^He dilution refrigerator with a nominal base temperature of 120 mK. The measurements on Sample S1 have been performed at this temperature. Samples S2–S5 have been measured at 14 mK to 1.5 K, see Figure S1 (Supporting Information).

The *R*_*xx*_ and *R*_*xy*_ data have been obtained directly from dividing the appropriate voltages and currents measured by the lock‐in amplifiers. No symmetrization or background subtraction have been performed.

## Conflict of Interest

The authors declare no conflict of interest.

## Supporting information

Supporting Information

## Data Availability

The data that support the findings of this study are available from the corresponding author upon reasonable request.
