# Supplementary material for: Spectral Asymmetry Induces a Re‐Entrant Quantum Hall Effect in a Topological Insulator
Source: Adv Sci (Weinh). 2024 Mar 13;11(19):2307447. doi: 10.1002/advs.202307447 (PMC11109608; doi:10.1002/advs.202307447)
Supplement: Supplementary file 1 — Supporting Information [file ADVS-11-2307447-s001.pdf]

ADVANCED SCIENCE
Open Access

Supporting Information

for *Adv. Sci.*, DOI 10.1002/advs.202307447

Spectral Asymmetry Induces a Re-Entrant Quantum Hall Effect in a Topological Insulator

Li-Xian Wang, Wouter Beugeling*, Fabian Schmitt, Lukas Lunczer, Julian-Benedikt Mayer, Hartmut Buhmann, Ewelina M. Hankiewicz and Laurens W. Molenkamp**

Supporting Information

Spectral asymmetry induces a re-entrant quantum Hall effect in a topological insulator

Li-Xian Wang*, Wouter Beugeling*, Fabian Schmitt, Lukas Lunczer, Julian-Benedikt Mayer, Hartmut Buhmann, Ewelina M. Hankiewicz, and Laurens W. Molenkamp*

NOTE S1: ADDITIONAL SAMPLES

Device label	Substrate	Layer stack	Mn concentration x	Tensile strain $(\%)$
S1 (presented)	Si-doped GaAs	15 nm $Hg_{1-y}Cd_yTe$<br>73 nm $Hg_{1-x}Mn_xTe$<br>100 nm $Hg_{1-y}Cd_yTe$	0.017	0.36
S2	Si-doped GaAs	14 nm $Hg_{1-y}Cd_yTe$<br>81 nm $Hg_{1-x}Mn_xTe$<br>100 nm $Hg_{1-y}Cd_yTe$	0.011	0.35
S3	CdTe	11.5 nm $Hg_{1-y}Cd_yTe$<br>64 nm $Hg_{1-x}Mn_xTe$<br>103 nm $Hg_{1-y}Cd_yTe$	0.031	0.39
S4	CdTe	14 nm $Hg_{1-y}Cd_yTe$<br>74 nm $Hg_{1-x}Mn_xTe$<br>100 nm $Hg_{1-y}Cd_yTe$	0.017	0.34
S5	CdTe	92 nm $Hg_{1-x}Mn_xTe$	0.044	0.39

TABLE S1. **Measured $Hg_{1-x}Mn_xTe$ samples with varying substrate, layer stacks, Mn concentration, and strain.** The presented sample is labelled "S1", and the rest are labelled from "S2" to "S5", respectively. The column "Layer stack" indicates the relevant layer thicknesses and materials. Samples S1–S4 have $Hg_{1-y}Cd_yTe$ barriers with Cd concentration $y = 0.68$. In sample S5, the $Hg_{1-x}Mn_xTe$ layer was grown directly onto the substrate. The measured samples all exhibit re-entrant quantum Hall effect at low temperatures, see Figure S1.

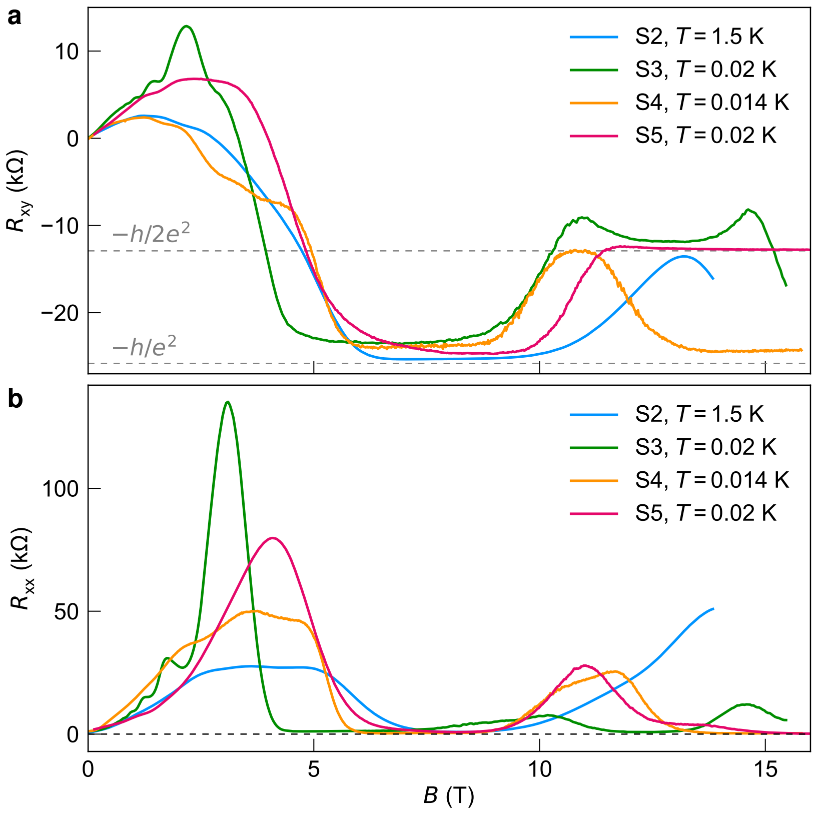

FIGURE S1. **Four further samples with varying Mn concentration and thickness.** a) Hall resistance R_{xy} and b) Longitudinal resistance R_{xx} as function of magnetic field B. The measurement temperature T of each curve is also labelled. For detailed information for each sample, refer to Table S1.

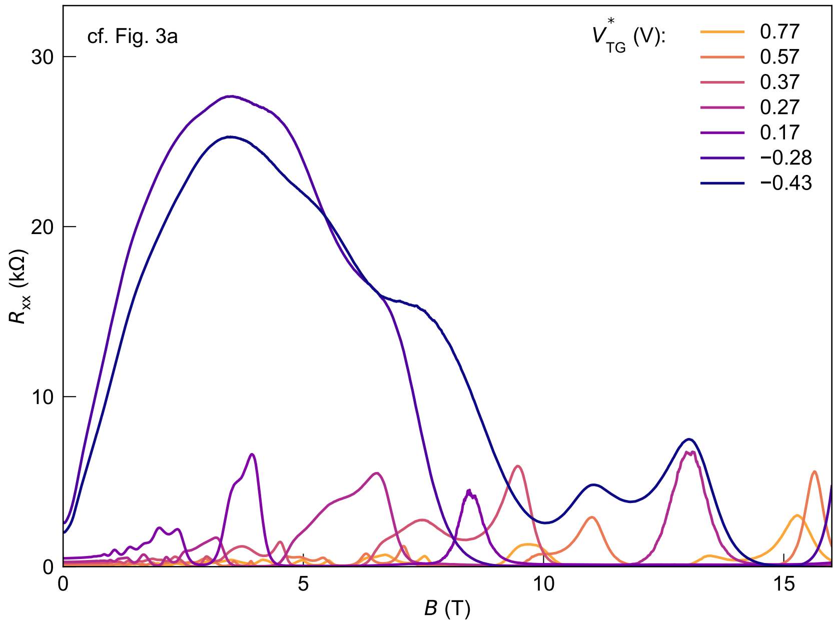

FIGURE S2. **Longitudinal resistance for a 73 nm thick (Hg,Mn)Te layer.** The longitudinal resistance R_{xx} for sample S1, for several values of the top gate voltage V^*_{TG}. This data has been measured simultaneously with R_{xy} in Figure 3a (main text).

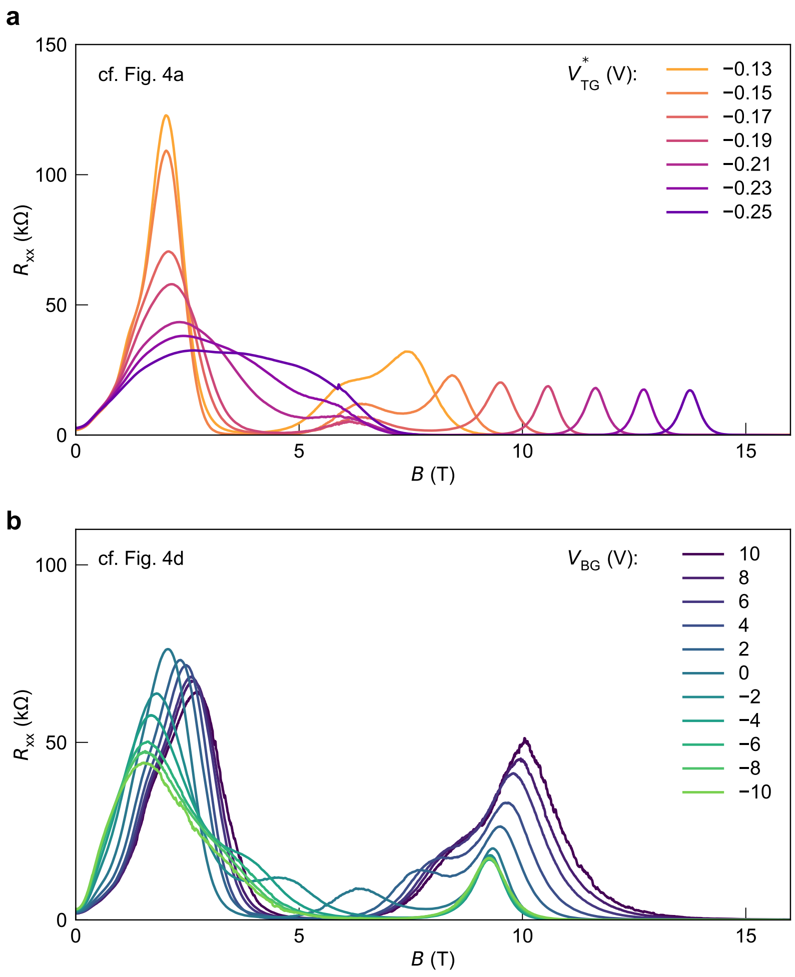

FIGURE S3. **Longitudinal resistance in the re-entrance regime.** a) The longitudinal resistance R_{xx} for sample S1, for various top gate voltages V^*_{TG}. This data has been measured simultaneously with R_{xy} in Figure 4a (main text) b) The longitudinal resistance R_{xx} for sample S1, for various bottom gate voltages V_{BG}. This data has been measured simultaneously with R_{xy} in Figure 4d (main text).

NOTE S2: EXTRACTION OF ELECTRON AND HOLE DENSITIES

The magneto-transport data of Figures 1b and 4a (main text) shows a clear two carrier behavior for negative top gate voltages. The n-type carriers with high mobility dominate the low field transport, and the p-type carriers dominate at high fields due to a lower mobility. The slope of the Hall resistance at low magnetic fields (see Figure S4a) can be related to the n-type carrier density n_e by $n_\mathrm{e} = 1/(e\, dR_{xy}/dB)$, where e is the elementary charge. In the p-type dominated regime, at high magnetic fields, the quantum Hall effect is already well developed. We extract the field B' of the transition from the $\nu = -2$ to the $\nu = -1$ plateau at high fields, which comes purely from the p-type carriers. For increased accuracy, we determine B' from the corresponding maximum of R_{xx}, see Figure S4b. Thus, we find the p-type density as $n_\mathrm{h} = -\overline{\nu} B' e/h$ with $\overline{\nu} = -\frac{3}{2}$.

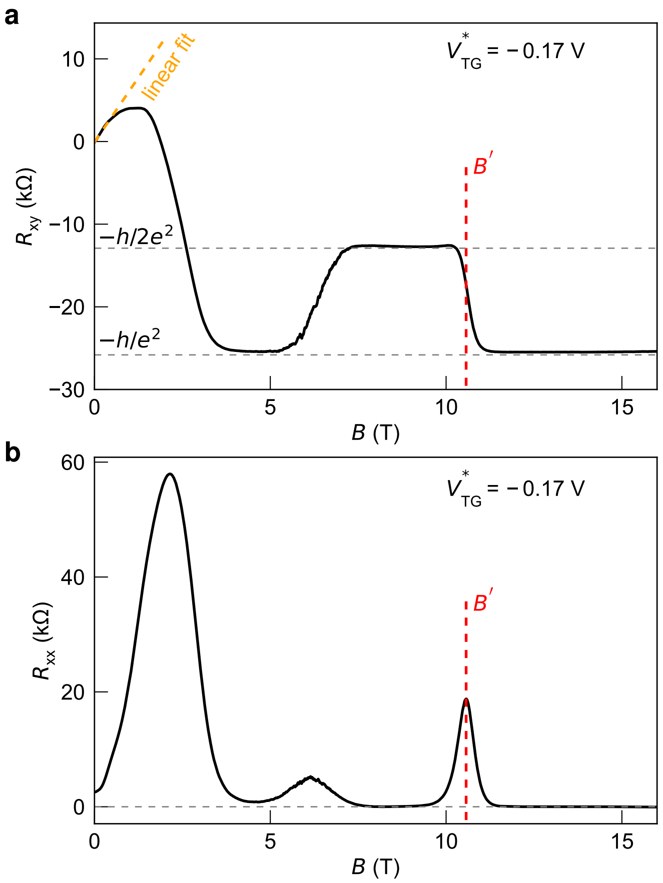

FIGURE S4. **Extraction of electron and hole densities.** a) Hall resistance R_{xy} for $V^*_{\mathrm{TG}} = -0.17$ V, replotted from Figure 1b (main text). The slope of the linear fit at small magnetic fields is used to determine the n-type density n_{e}. We indicate the field B' at the transition between the $\nu = -2$ and $\nu = -1$ plateaus at high magnetic fields, from which we extract the p-type density n_{h}. b) The value B' is defined by the maximum of R_{xx} corresponding to this transition.

NOTE S3: LOW FIELD QUANTUM HALL PLATEAU

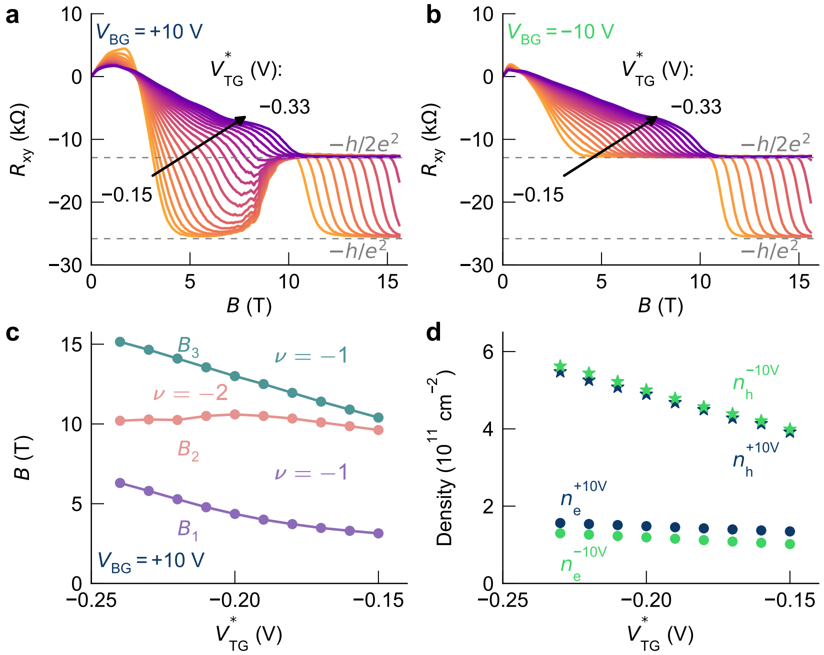

FIGURE S5. **Low-field $\nu = -1$ quantum Hall plateau and its relation to the bottom surface state.** a) Hall resistance R_{xy} as a function of magnetic field from $V^*_{\rm TG} = -0.15$ V to -0.33 V at $V_{\rm BG} = +10$ V. b) R_{xy} as a function of magnetic field from $V^*_{\rm TG} = -0.15$ V to -0.33 V at $V_{\rm BG} = -10$ V. $R_{xy} = -h/e^2, -2h/e^2$ are indicated by dashed lines. c) Characteristic fields extracted from a) for $V^*_{\rm TG} = -0.24$ V to -0.15 V. We have indicated the quantum Hall filling factors ν between the characteristic fields. d) Electron density for $V_{\rm BG} = +10$ V ($n_{\rm e}^{+10V}$) and -10 V ($n_{\rm e}^{-10V}$) and hole density for $V_{\rm BG} = +10$ V ($n_{\rm h}^{+10V}$) and -10 V ($n_{\rm h}^{-10V}$) for $V^*_{\rm TG} = -0.23$ V to -0.15 V.

NOTE S4: CALCULATION OF EXPERIMENTALLY RELEVANT LANDAU FAN

In this section, we introduce the method used to calculate the Landau level fan chart displayed in the main text (Figure 5). Conventional fans charts display the energy dependence of each Landau level. However, in actual magneto-transport experiments, one obtains Hall conductance as a function of gate voltage rather than energy. Changing the gate voltage not only varies the total carrier density $n_{\mathrm{tot}} = n_{\mathrm{e}} - n_{\mathrm{h}}$, but also the electrostatic potential.

In order to model the effect of the electrostatics on the band structure, we add a Hartree potential $U_{\mathrm{H}}(z)$ to the 8-orbital $k \cdot p$ Hamiltonian (for the latter, see e.g., Ref. [1]). The Hartree potential is a function of position z in the growth direction only, and its functional dependence is determined by the carrier densities on the top and bottom surfaces. We construct it as a sum of two potential functions, $U_{\mathrm{H,T}}(z)$ and $U_{\mathrm{H,B}}(z)$, related to charges at the top and bottom surfaces, respectively, as illustrated in Figure S6a. Assuming uniform charge densities within a distance $d_{\mathrm{s}} = 8$ nm from the interfaces [2], $U_{\mathrm{H,T}}(z)$ and $U_{\mathrm{H,B}}(z)$ show a quadratic functional dependence near the top ($z = d_{\mathrm{TI}}/2$) and bottom ($z = -d_{\mathrm{TI}}/2$) interfaces, respectively, and vanish in the bulk. We assign the value of potential functions at the interfaces as $U_{\mathrm{T}} \equiv U_{\mathrm{H,T}}(d_{\mathrm{TI}}/2)$ and $U_{\mathrm{B}} \equiv U_{\mathrm{H,B}}(-d_{\mathrm{TI}}/2)$. Thus, $U_{\mathrm{H}}(z)$ is determined by three parameters: U_{T}, U_{B}, and the thickness of surface-state region $d_{\mathrm{s}} = 8$ nm. The total carrier density n_{tot} relates to these parameters as

$$n_{\mathrm{tot}} = \frac{\epsilon_{\mathrm{r}}\epsilon_0}{ed_{\mathrm{s}}}(U_{\mathrm{T}} + U_{\mathrm{B}}) \tag{S1}$$

where ϵ_{r} is the dielectric constant at the surface state region [2].

In order to simulate the effect of a changing top gate, we only vary U_{T} and keep U_{B} constant. This is justified by the screening of the gate electric field by the surface states [3]. For each given U_{H}, we perform $k \cdot p$ calculations to obtain Landau levels as a function of magnetic field B and energy E. For total density $n_{\mathrm{tot}} = 2.2 \times 10^{11}\ \mathrm{cm}^{-2}$ and $n_{\mathrm{tot}} = -1.8 \times 10^{11}\ \mathrm{cm}^{-2}$ (corresponding to the densities shown in Figure 5 of the main text), we illustrate the Landau fan diagrams in Figures S6b and S6d, respectively. (As input for the Hartree potential U_{H}(z), we have used $U_{\mathrm{T}} = -18$ meV, $U_{\mathrm{B}} = -30$ meV, and $U_{\mathrm{T}} = +69$ meV, $U_{\mathrm{B}} = -30$ meV, respectively.) For each Landau level dispersion $E(B)$, we calculate the corresponding carrier density $n_{\mathrm{tot}}(B)$, and thus map the energy-versus-field Landau fan to a density-versus-field fan, as shown for Figures S6c and S6e for the aforementioned densities.

From the density-versus-field plot, we can readily extract the positions of the Landau levels by finding the intersections of the Landau level dispersion with constant density n_{tot}, i.e., the

yellow and black horizontal lines in Figures S6c and S6e, respectively. (In the energy-versus-field diagrams, Figures S6b and S6d, the Fermi energy as function of magnetic field is indicated as the yellow and black curves, respectively.) The values where the intersection points occur determine the locations of the Landau transitions in the $\sigma_{xy}(B)$ dependence shown in Figures 5a and 5c of the main text. By sweeping through intermediate density values n_{tot}, we obtain Figure 5b of the main text. Some Landau levels may appear at more than one density value n_{tot}. This remarkable behaviour occurs due to the intricate non-monotonic dependence of the Fermi level as function of n_{tot} which results from pinning to the massive surface state [2].

Complementary to the location of the wave function (expectation value $\langle z \rangle$) indicated in Figure 5b (main text) as blue and red color, we also provide the orbital character of Landau levels in Figure S7. The Landau level labelled "mass." from the massive surface state originates from the $\Gamma_{8,\pm 3/2}$ orbitals, and the "zero LL" Landau level is from the $\Gamma_{8,\pm 1/2}$ orbitals, manifesting the band inversion at lower fields and its restoration to normal band ordering at higher fields.

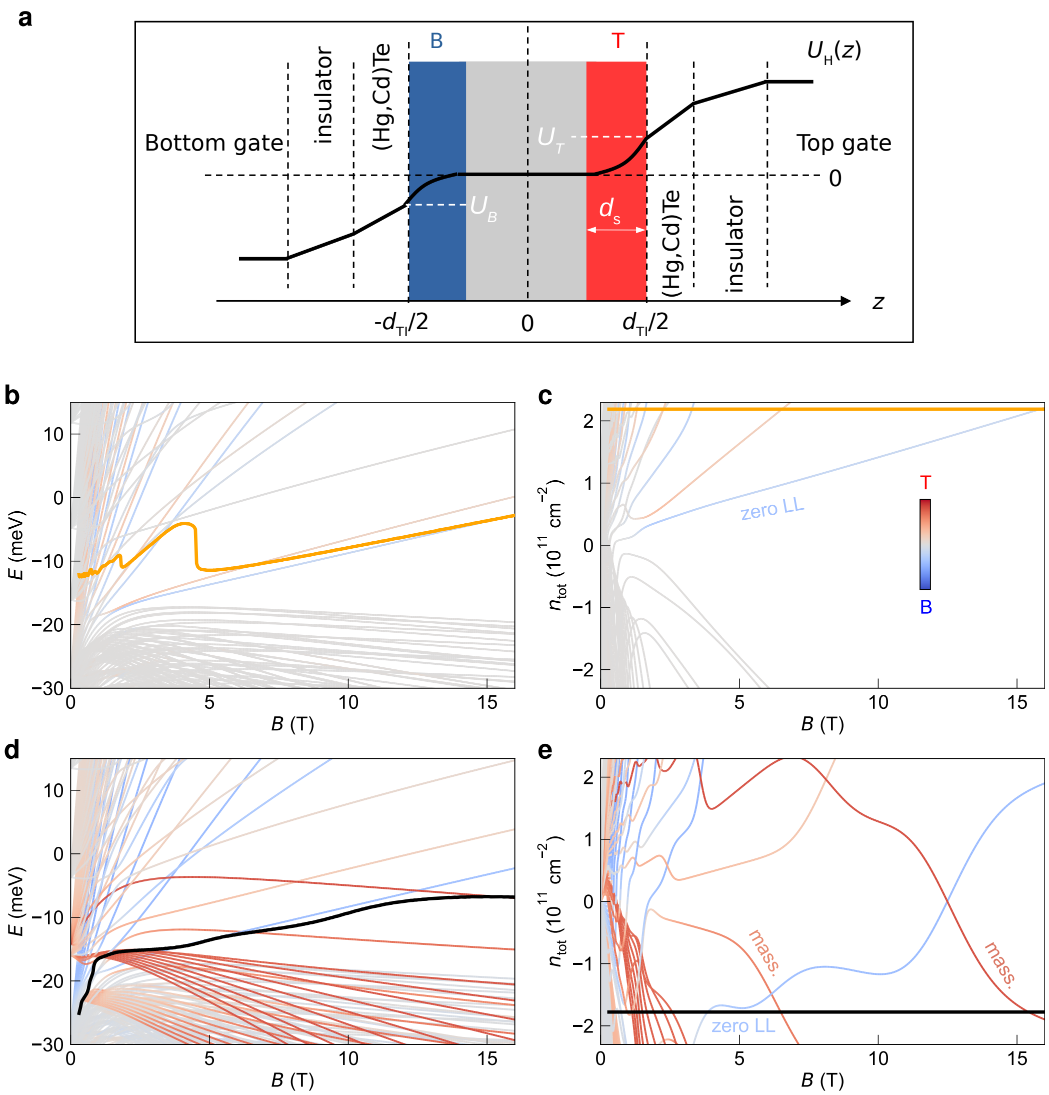

FIGURE S6. **Landau level calculations for positive and negative top gate voltage.** a) Sketch of Hartree potential $U_\mathrm{H}(z)$ as a function of position z in crystal growth direction. U_T and U_B indicate the Hartree potential at the top and bottom interfaces, respectively. d_s stands for thickness of surface-state region (labelled T and B for top and bottom surface, respectively). b) Energy E versus magnetic field B plot and c) total electron density n_tot versus B plot of Landau levels for $n_\mathrm{tot} = 2.2 \times 10^{11}$ cm^{-2} ($V^*_\mathrm{TG} \approx 0.1$ V), corresponding to the yellow line in Figure 5b (main text). d) E versus B plot and e) n_tot versus B plot of Landau levels for $n_\mathrm{tot} = -1.8 \times 10^{11}$ cm^{-2} ($V^*_\mathrm{TG} \approx -0.2$ V; black line in Figure 5b).

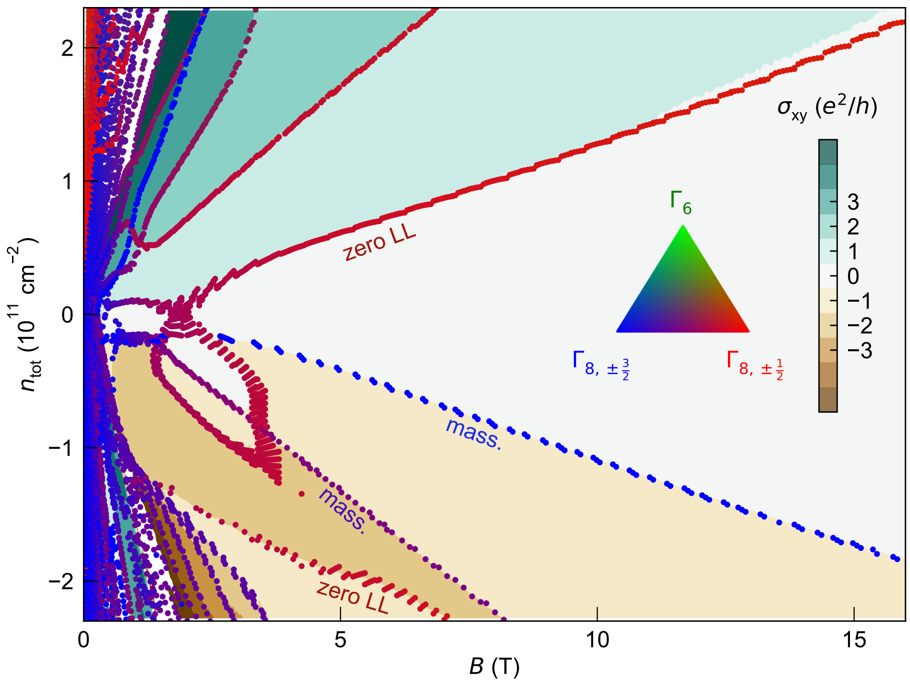

FIGURE S7. **Orbital character of calculated Landau level fan.** The displayed result is from the same calculations performed for Figure 5b (main text). The color code in the background is associated with the Hall conductivity σ_{xy} in unit of e^2/h. The Landau levels are indicated by the dots, the color of which (legend in inset) indicates their orbital character (a combination of Γ_6, $\Gamma_{8,\pm1/2}$ and $\Gamma_{8,\pm3/2}$ orbitals). Note that the data at small fields and high $|n_{\rm tot}|$ (shaded region) is inaccurate due to limitations of the Landau level calculations.

NOTE S5: THEORY OF SPECTRAL ASYMMETRY FOR A SINGLE SURFACE STATE

To analyze the contribution of a single surface state to σ_{xy}, we use an effective Hamiltonian in the basis $\{|\mathrm{B};\uparrow\rangle, |\mathrm{B};\downarrow\rangle\}$ of the two spin components of the bottom surface state,

$$H_{\mathrm{B}} = \mathcal{C}\sigma_0 + m_k\sigma_z - \mathcal{A}(k_y\sigma_x - k_x\sigma_y) \tag{S2}$$

where $\sigma_{x,y,z}$ is a set of Pauli matrices acting on the spin degree of freedom and $m_k = \mathcal{M} - \mathcal{B}|k|^2$ is an effective mass term (written up to second order in $|k|$) which needs to appear to cure the parity anomaly. In contrast to a single block of the BHZ model [4], the σ_z terms of this Hamiltonian break time-reversal symmetry. The Dirac mass m_k can only be non-zero in a finite magnetic field; for $B = 0$, $\mathcal{M} = \mathcal{B} = 0$. In our system, $\mathcal{M}$ is realized by a combination of a Zeeman field, the exchange coupling generated by the Mn doping, and the orbital field, that originates from the Peierls substitution. The quadratic $\mathcal{B}$ term arises from the coupling of the surface state with the bulk $j_z = \pm 3/2$ states in a magnetic field, and satisfies $\mathcal{B} < 0$.

We are interested in the evolution of the surface state in the presence of a magnetic field. Applying the Peierls substitution in the Landau gauge $\mathbf{k} \to \mathbf{k} + e\mathbf{A}/\hbar$ with $\mathbf{A} = -yB\mathbf{e}_x$, we obtain the Landau level structure by replacing the canonical momentum operators with ladder operators [5]

$$E^{\pm}_{n\neq 0} = \mathcal{C} + s\frac{\beta}{2} \pm \sqrt{n\alpha^2 + [\mathcal{M} - n\beta]^2} \tag{S3}$$

$$E_{n=0} = \mathcal{C} - s\left[\mathcal{M} - \frac{\beta}{2}\right] \tag{S4}$$

where $s = \mathrm{sgn}(eB)$ is the direction of the magnetic field, $\beta = 2\mathcal{B}/l_B^2$, $\alpha = \sqrt{2}\mathcal{A}/l_B$, $l_B^2 = \hbar/|eB|$, and n is the Landau level index. The surface state forms a Landau level fan with a single decoupled $n = 0$ Landau level.

The single Landau level with $n = 0$ lies either above or below the charge neutrality point, creating an imbalance between the number of states above or below, i.e., a spectral asymmetry. Intuitively, the spectral asymmetry can be calculated by counting the Landau level states above and below the charge neutrality point E_z [6, 7],

$$\eta_B = \sum_{E>E_z} 1 - \sum_{E<E_z} 1. \tag{S5}$$

Here, the charge neutrality point is at $E_z = \mathcal{C}$, which is determined by demanding a vanishing particle number in the ground state [8]. In view of the divergent sums of Equation (S5), we apply a heat-kernel regularization following Ref. [7] in order to make η_B well-defined.

By definition, η_B only changes when a Landau level crosses the charge neutrality point as one varies the magnetic field. From Equation (S3), one can see that the $n \neq 0$ Landau levels never do so. Only the $n = 0$ state can cross E_z, i.e., whenever $B = s\hbar\mathcal{M}/(e\mathcal{B})$ is satisfied. This results in a jump of ± 2 in the spectral asymmetry. We apply the method used in Ref. [7] and obtain

$$\eta_B = -s \left[\mathrm{sgn}(\mathcal{B}) + \mathrm{sgn}\left(\mathcal{M} - \frac{\mathcal{B}}{l_B^2} \right) \right]. \tag{S6}$$

The contribution $\sigma_{xy}^{\mathrm{BSS}}$ of the bottom surface state to the Hall conductance at charge neutrality can be evaluated as

$$\sigma_{xy}^{\mathrm{B}} = -\frac{e^2}{2h} s \left[\mathrm{sgn}(\mathcal{B}) + \mathrm{sgn}\left(\mathcal{M} - \frac{\mathcal{B}}{l_B^2} \right) \right] \tag{S7}$$

from a calculation of the Chern number for H_{B}. This contribution relates to the spectral asymmetry as $\sigma_{xy}^{\mathrm{B}} = (e^2/h)(\eta_B/2)$, and can take discrete values depending on the competition between $\mathcal{M}$ and $\mathcal{B}/l_B^2$. If the signs of both terms in Equation (S7) are equal, we find a finite contribution of $\pm e^2/h$ to the total σ_{xy} due to the parity anomaly. If the terms have opposite sign, the contribution vanishes.

The above is a minimal model with two degrees of freedom, to illustrate the effect of the spectral asymmetry on σ_{xy}. The principle remains valid for more realistic band structures with many more degrees of freedom taken into account, e.g., the bulk valence band states and the massive surface states at the opposite surface. In the main text, we have retrieved the signature of spectral asymmetry in a full $k \cdot p$ model.

---

[1] E. G. Novik, A. Pfeuffer-Jeschke, T. Jungwirth, V. Latussek, C. R. Becker, G. Landwehr, H. Buhmann, L. W. Molenkamp, *Phys. Rev. B* **2005**, *72*, 035321.

[2] D. M. Mahler, V. L. Müller, C. Thienel, J. Wiedenmann, W. Beugeling, H. Buhmann, L. W. Molenkamp, *Nano Lett.* **2021**, *21*, 9869.

[3] C. Brüne, C. Thienel, M. Stuiber, J. Böttcher, H. Buhmann, E. G. Novik, C.-X. Liu, E. M. Hankiewicz, L. W. Molenkamp, *Phys. Rev. X* **2014**, *4*, 041045.

[4] B. A. Bernevig, T. L. Hughes, S.-C. Zhang, *Science* **2006**, *314*, 1757.

[5] M. König, H. Buhmann, L. W. Molenkamp, T. Hughes, C.-X. Liu, X.-L. Qi, S.-C. Zhang, *J. Phys. Soc. Jpn* **2008**, *77*, 031007.

[6] A. J. Niemi, G. W. Semenoff, *Phys. Rev. Lett.* **1983**, *51*, 2077.

[7] J. Böttcher, C. Tutschku, L. W. Molenkamp, E. M. Hankiewicz, *Phys. Rev. Lett.* **2019**, *123*, 226602.

[8] J. Böttcher, C. Tutschku, E. M. Hankiewicz, *Phys. Rev. B* **2020**, *101*, 195433.
